# Impact of Music Therapy on Patients in the Critical Care Unit: A Qualitative Study

**DOI:** 10.1111/nicc.70099

**Published:** 2025-06-26

**Authors:** Verónica Saldaña‐Ortiz, Ana Recio‐Rivas, José Miguel Mansilla‐Domínguez, Esther Martínez‐Miguel

**Affiliations:** ^1^ Department of Nursing, Faculty of Medicine, Health and Sports European University of Madrid Madrid Spain; ^2^ Director of Nursing Degree, Faculty of Life and Natural Sciences Nebrija University Madrid Spain

**Keywords:** critical care, music therapy, patients, recovery, relaxation

## Abstract

**Background:**

Music therapy is the use of music by a trained professional to help people improve their health, emotions or well‐being. It can involve listening to music, playing instruments, singing or writing songs as part of therapy. In intensive care settings, music therapy plays a pivotal role in patient‐centred care. This study delves into the experiences of critically ill patients, underscoring music's capacity to evoke emotions and transcend linguistic barriers. This study delves into music therapy as a promising intervention that alleviates stress, fosters emotional expression and enhances patients' quality of life.

**Aim:**

The aim of this study is to find out what the intensive care patient's experience is like during a music therapy session and their perception of its influence on their disease process and subsequent recovery.

**Study Design:**

This study employed a descriptive qualitative approach. A qualified music therapist conducted a 20‐min music therapy session with a sample of 14 patients in the Critical Care Unit. Data were collected through semi‐structured interviews.

**Results:**

After analysis of the results, three main themes have emerged: (1) humanising and accompanying the critical care experience; (2) music therapy as a form of relaxation; and (3) relief and recovery through music therapy.

**Conclusions:**

Patients in the intensive care unit (ICU) often feel stressed, vulnerable and isolated from their everyday environment. Music therapy offers a temporary escape, providing comfort and emotional connection and helping to reduce stress and anxiety. This therapy can awaken memories and emotions, stimulating cognition and facilitating emotional expression, which is crucial for patients with confusion or cognitive difficulties. In addition, it acts as an effective distraction from pain, improving mood and response to treatments, thus benefiting emotional recovery and coping.

**Relevance to Clinical Practice:**

This study advocates for the integration of music therapy programmes in the ICU, emphasising their benefits in reducing stress, alleviating pain and enhancing emotional well‐being. The findings contribute to the development of protocols with specific recommendations for its effective implementation. Furthermore, the study highlights the crucial role of nurses as key facilitators in enabling the incorporation of music therapists into the healthcare team through an interdisciplinary approach. Their involvement promotes collaboration among professionals and encourages the use of non‐pharmacological interventions. Additionally, it underscores the importance of nurse participation in the creation of protocols to ensure their applicability and effectiveness in clinical practice. The research supports music therapy as a valuable complementary approach in intensive care.

**Summary**

- What is known about this topic
  ○ Music therapy enhances non‐verbal communication and emotional connection between critically ill patients and their families.
  ○ It supports clinical goals while also helping to humanise care in complex healthcare settings.
  ○ The approach highlights the role of emotional well‐being in the overall recovery process.
- What this paper adds
  ○ Music therapy in intensive care helps humanise care, reduce stress and ease feelings of isolation.
  ○ It fosters emotional expression and strengthens bonds between patients, families and healthcare staff, supporting psychological well‐being.
  ○ By evoking memories and offering distraction from pain, music enhances emotional recovery and improves treatment outcomes, highlighting the value of complementary therapies in clinical practice.

## 1 Introduction

The intensive care unit (ICU) is defined as an organisation of healthcare professionals providing multidisciplinary care in a specific area of the hospital, caring for patients who require respiratory support together with support for at least two organs or systems, as well as all complex patients requiring support due to multi‐organ failure [1]. One of the main consequences of the ICU environment and the stressors experienced during the stay is the well‐documented Post Intensive Care Stress Syndrome (PICS). Patients treated in the ICU or with prolonged ward stays may have sequelae that require specialised rehabilitation. A considerable proportion of these patients also require initial transfer to a neurorehabilitation centre within the hospital setting to recover from moderate to severe sequelae. This involves specific therapies in the fields of physiotherapy, occupational therapy, speech therapy and neuropsychology [2]. Over the past 15 years, studies of cognitive, physical and mental function among survivors of critical illness have indicated that a significant proportion of these patients suffer from new or worsening impairments and disabilities. This has led to the concept of PICS being proposed [3].

The role of nursing professionals in relation to the care of the person and family members is of great importance. They maintain direct and constant contact with them, which allows them to help them to calm down and/or alleviate feelings such as stress, anxiety, uncertainty and anguish [4]. Nurses in an adult ICU have a pivotal role in the diagnosis, intervention and pursuit of solutions to stress factors. This is in accordance with the objective of providing humane and comprehensive care, which facilitates the establishment of emotional bonds and minimises unpleasant feelings in hospitalised individuals and their families [5].

In Spain, the “HUCI Project” is the “Manual of Good Practices of Humanisation in Intensive Care Units,” which is based on seven strategic lines for its development in the ICU [6]. Strategic line number seven for the humanisation of care, entitled “Humanised infrastructure”, incorporates complementary therapies into the healthcare system. The prevailing focus of conventional medicine on treating and supporting potentially reversible conditions with the objective of survival can frequently result in the dehumanisation of the ICU [7]. The integration of complementary therapies into conventional medical practice can enhance the patient experience. It is evident that music‐ and animal‐assisted interventions have the potential to enhance the psychophysiological condition of patients, thereby offering a more human‐centred approach [7].

The potential psychological benefits of physical rehabilitation in the ICU have been considered due to the well‐documented link between physical and mental health. These psychological benefits are greater when a ‘humanised’ or person‐centred approach is adopted. This is consistent with the findings of the study by Laerkner et al. [8], which described that ICU patients found ‘hope through mobilisation’ [8]. It can be reasonably assumed that anxiety is a common occurrence in patients who are experiencing neurocritical illness, as well as in their primary caregivers.

As the number of patients surviving intensive care increases, mental health problems have become a subject of growing interest for researchers. Among these, post‐traumatic stress disorder can have a significant impact on the quality of life of intensive care survivors. A study by Righy et al. [9] of cognitive, physical and mental function among critically ill patients found that a significant proportion of these patients suffered from new or worsening impairments and disabilities, which gave rise to the previously discussed concept (SPCI) [9].

It can be posited that music therapy, as defined by Wigram et al. [10], is designed to facilitate the development of an individual's potential and/or restoration of impaired functions, thereby enabling them to achieve both intra‐ and interpersonal integration and, as a consequence, a superior quality of life. This can be achieved through the implementation of prevention, rehabilitation or treatment strategies [10].

Music therapy is a therapeutic approach that uses music to promote overall health and well‐being. It may involve composing, singing, movement, listening or relaxation techniques. Recognised as a form of complementary medicine, it helps alleviate pain, anxiety, depression and stress related to illnesses such as cancer and its treatment [11]. In his 1997 book, “Defining Music Therapy”, Bruscia, an authority on the use of music as therapy, posits that music therapy is “transdisciplinary”. This implies that it is not confined to a single isolated discipline with clear and immutable boundaries; instead, it is a dynamic combination of multiple disciplines encompassing two main areas: music and therapy. The convergence of music‐related disciplines with therapy‐related disciplines results in the formation of a hybrid discipline known as music therapy [12].

Finally, the team led by Greenberg et al. [13] emphasises the significance of oxytocin and neurocircuits related to reward, stress and the immune system. The study demonstrated that the social brain networks associated with music production, as opposed to those involved in music listening, are analogous to networks associated with social processes in human cognition, such as mentalisation, empathy and synchrony. Moreover, these components have evolved to foster social affiliation and connectivity [13].

## 2 Justification for the Study

The present research is motivated by the necessity to explore and comprehend the subjective experiences of critically ill patients participating in music therapy sessions. In a hospital setting, particularly in Critical Care Units (CCUs), the primary focus is on the clinical management of acute conditions. However, there is an increasing recognition of the benefits of non‐pharmacological interventions [14, 15], such as music therapy, on the emotional, psychological and physical well‐being of patients.

While numerous studies have demonstrated the positive effects of music therapy, such as the reduction of stress [16], depression [17] and pain, there is limited evidence available that explores patients' personal experiences and perceptions of its influence on the disease process and their recovery [18]. This is particularly salient in the context of critical care, wherein patients frequently encounter circumstances of heightened vulnerability, uncertainty and distress.

The present study adopts a descriptive qualitative design, enabling a qualitative approach to capture the lived experiences of patients and offering a rich and detailed perspective on their perception of the music intervention. Comprehension of these experiences enhances knowledge regarding the perceived impact of music therapy and also furnishes valuable information to personalise and optimise interventions in critical contexts.

Furthermore, the integration of music therapy as part of integrated care strategies in CCUs may be furthered, supporting its inclusion in evidence‐based guidelines and protocols. The ultimate objective is to furnish healthcare professionals with supplementary instruments to address the biopsychosocial needs of critically ill patients, enhancing not only their physical recovery but also their quality of life during hospitalisation.

Consequently, this research not only addresses a gap in knowledge, but also has practical and ethical implications for humanised care in the critical care setting.

## 3 Aim of the Study

The aim of this study is to find out what the patient's experience is like during a music therapy session and their perception of its influence on their disease process and subsequent recovery.

## 4 Design and Methods

This study adopted a descriptive qualitative design to explore participants' experiences in a straightforward and context‐sensitive manner. Qualitative descriptive research produces data that captures the “who, what and where” of experiences or events from the participants' subjective viewpoint. This design is particularly suitable when direct information is needed from those who have experienced the phenomenon under study [19].

Music therapy was implemented by a professional known as a music therapist. In our context, a professional known as a music therapist holds a degree in Musicology, followed by a specialised master's degree in music therapy. This academic and professional training provides the necessary competencies to apply music‐based interventions in clinical settings.

The music therapy intervention was designed to be personalised and patient‐centred. Before the session, the therapist consulted with family members or caregivers to determine the patient's musical preferences, ensuring that the selected music would be meaningful and emotionally resonant for the individual. Sessions lasted an average of 20 min and were conducted simultaneously with invasive medical procedures, such as oral mucosal lavage, endotracheal tube extubation or central venous catheter management. During these sessions, the selection of songs was based on a comprehensive and individualised process. The music therapist engaged in discussions with family members, caregivers and, when possible, the patients themselves to gather information about their musical preferences, personal history and cultural background. Additionally, implicit reactions to different musical stimuli were observed by the music therapist during the sessions to further refine the selection. The goal was to ensure that the chosen music resonated with the patient on both an emotional and physiological level. This process allowed for the use of familiar and personally meaningful music to enhance engagement and therapeutic outcomes.

The music therapy sessions were conducted live, with the music therapist playing live instruments next to the patient, allowing for real‐time adaptation to each patient's needs and clinical situation. Personalised music was used, tailored to the individual characteristics of each patient. The intervention included the use of voice and musical instruments such as guitar and percussion. Additionally, the volume of the music was carefully adjusted according to the patient's breathing patterns and clinical condition to ensure a supportive and therapeutic environment.

The music used in the sessions cannot be generalised, as it was personalised according to each patient's preferences and clinical condition. The tempo, rhythmic structure, form, dynamics and lyrical content were adapted in real‐time to align with the therapeutic goals and the patient's physiological and emotional state. This individualised approach ensured that the music was either stimulating or sedating, depending on the needs of each patient.

### 4.1 Setting and Participants

The study was carried out in the Adult Intensive Care Unit of a second level hospital of the Community of Madrid, Spain. The Intensive Care Unit (ICU) where this study was conducted comprises 18 beds, four of which are designated for isolation. Music therapy sessions were conducted during visiting hours, which were 11:00–14:00 and 16:00–19:00. Visits were scheduled at either of the two designated times and no more than two family members were present in the designated ICU room.

The study's inclusion criteria required patients to be between 18 and 75 years old, oriented to person, time and space and able to maintain oral communication. Exclusion criteria included underlying psychiatric pathology and sedation. A purposive sampling approach was initially employed to select participants who met the predefined inclusion criteria, ensuring that each individual could provide relevant and meaningful insights into the research topic. As data collection and analysis progressed, theoretical sampling was then used to guide the selection of additional participants based on emerging concepts and categories. This allowed the researcher to explore and develop theoretical constructs in greater depth, selecting new participants who could help refine, expand or contrast these emerging themes.

The study sample was selected based on patient‐specific inclusion criteria. The study's group of patients reached a point of data saturation at number 14, indicating that sufficient data had been collected to gain a comprehensive understanding of the phenomenon under investigation.

### 4.2 Data Collection

Fourteen semi‐structured interviews with patients were used to collect data for this study (Table 1). Patients were interviewed after they had been discharged from the ICU to their ward, 24/48 h after the session, to allow them to absorb what they had experienced. This allowed for a more relaxed and trusting atmosphere between the interviewer and participant. The interviewer was the principal investigator of the study and was distinct from the music therapist. While the music therapist conducted the therapeutic sessions, the principal investigator, who was not involved in the direct therapeutic process, was responsible for conducting the interviews. This distinction ensured that the data collection was unbiased and separated from the therapeutic interventions.

**TABLE 1:** Interview guide.

Aspect to explore	Sample questions
Find out what the patient feels after listening to the music therapy session about his or her disease process	What do you feel while you are hospitalised in the Critical Care Unit while listening to music therapy? While listening to music, what do they think about?
To identify what perspective patients have of the ICU environment and the effect music therapy has on this environment.	What is their overall image of the Critical Care Unit? What is their perception of the atmosphere in the unit after music therapy?
Find out how the patient feels after listening to the music therapy session about their recovery	How do you think music helps you in your healing process? In what way? How do you think music affects you regarding your disease process?

In this case, the interviewer had a ‘script’ that listed the topics to be covered during the interview. However, the order in which the different topics were addressed and the way in which the questions were asked were left to the interviewer's discretion and judgement. Within a given topic, the interviewer approached the interview as he/she saw fit, asked questions as needed, explained meanings, requested clarification if a point was not understood, asked the interviewee to elaborate when necessary and established his/her own personal interviewing style [20].

### 4.3 Data Analysis

This study employed Braun and Clarke's reflective thematic analysis, a method of qualitative data analysis that involves identifying recurring patterns in a data set in order to recognise, analyse and report on themes. The approach is comprised of several stages: familiarisation with the data, coding, generation of themes, review of themes, definition and naming of themes and writing up [21]. The objective of this approach is to identify patterns and themes within the data set and subsequently interpret them in order to gain a deeper understanding of the phenomenon under investigation [22] (Table 2).

**TABLE 2:** Phases for the thematic analysis of Braun and Clarke [22].

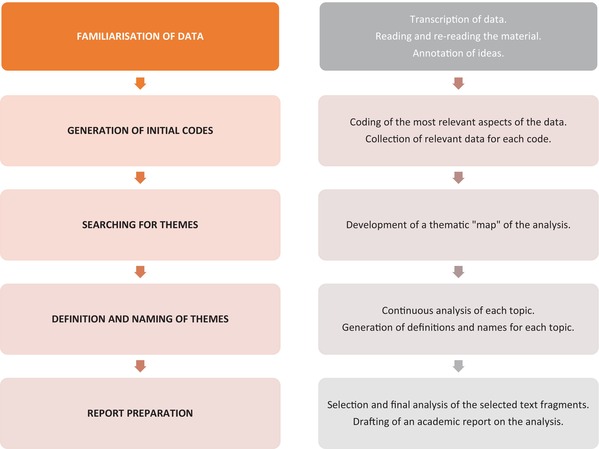

## 5 Ethical and Research Approvals

### 5.1 Ethical Considerations

The Consolidated Criteria for Reporting Qualitative Research (COREQ) in Appendix 1 has been used [23].

The study was formally endorsed on 25th April 2023 by the Ethics Committee of the European University of Madrid (EC 23.238).

The authors confirm that they have adhered to the protocols of their institution regarding the publication of participants' data. The right to privacy and informed consent has been respected throughout the course of this study.

The authors have obtained informed consent from participants throughout mentioned in the article, and this document is in the possession of the corresponding author.

This research has not received any form of financial or material support from public sector agencies, commercial organisations or non‐profit organisations. The authors declare that they have no conflicts of interest.

## 6 Results

In order to understand the results of this study, 14 semi‐structured interviews were conducted. The socio‐demographic data of the participants are explained in Table 3.

**TABLE 3:** Sociodemographic data.

Coding	Age	Sex	Level of education	Days of stay in ICU	Minutes of the session	Reason for admission	First time in ICU
PAT1	71	Woman	Basic	9	20	Acute pulmonary oedema	YES
PAT2	70	Woman	Basic	10	20	Acute pulmonary oedema	YES
PAT3	50	Man	Secondary Education	2	15	Hypovolemic shock, haemorrhage	NO
PAT4	65	Man	Basic	21	15	Acute myocardial infarction	YES
PAT5	72	Man	Basic	4	15	Acute myocardial infarction	YES
PAT6	63	Man	Basic	12	15	Hypovolemic shock	YES
PAT7	52	Woman	Basic	30	15	Pneumonia	YES
PAT8	18	Woman	Bachelor's Degree	60	15	Pneumonia	YES
PAT9	30	Woman	Bachelor's Degree	7	15	Surgical intervention	YES
PAT10	39	Woman	Psychologist	1	20	HIPEC	YES
PAT11	65	Man	Bachelor's Degree	16	20	Peritonitis	NO
PAT12	54	Man	Basic	50	15	Chicken bone choking	YES
PAT13	43	Woman	Basic	5	15	Malignant hyperthermia	YES
PAT14	75	Man	Basic	10	15	Ruptured spleen	YES
MEDIA	**73**			**16,92857143**	**16,42857143**		

*Note:* Bold indicates significant values’. Total number of days each patient spent in the Intensive Care Unit (ICU) and the duration, in minutes, of the music therapy sessions received during their stay. Important to note because of the significant relationship between length of stay and music therapy intervention.

Abbreviations: HIPEC: hyperthermic intraperitoneal chemotherapy; PAT: patient.

The following topics concern the experience of patients admitted to the Intensive Care Unit in relation to the music therapy session they received. The patients' experience is a consequence of the humanisation of care and the accompanying and proximate presence during the session. The patients described their perceptions of music therapy as a positive experience, a form of distraction and accompaniment during their illness. Furthermore, the patients indicate that music therapy facilitates closer relationships with their relatives and healthcare staff.

Patients report that participation in music therapy can alter their mood, whether they are experiencing a period of sadness or a more positive emotional state. This leads the patients to express that their experience during the sessions has been pleasant and enjoyable.

The music therapy sessions they received in the ICU helped them to distract themselves from the situation they were going through, focusing on the music they were listening to. This allowed them to meditate and have a moment of disconnection and introspection with themselves.

From the analysis of the content extracted from the face‐to‐face interviews, 3 themes emerged that represent the bulk of the experiences and emotions of the 14 study participants after the music therapy sessions. The main findings are described below, organised according to each theme:

### 6.1 Humanising Care Through Interdisciplinary Approaches in Critical Care

**FIGURE 1:**
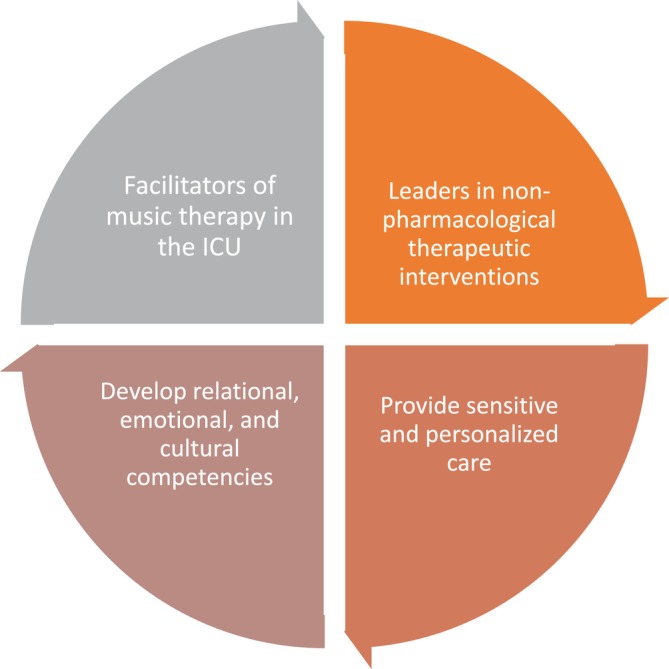
Nursing role in the integration of music therapy in critical care.

This theme refers to the beneficial and unusual aspect of music therapy in the ICU, which is described as an unforgettable event that has the effect of making the unit more humanised and closer to the patients and their families. Patients suggest that the music therapy sessions should be extended in duration to allow for a more comprehensive experience and indicate that this therapy could be generalised throughout the hospital due to its significant contribution to the patients and their families. It was posited by the patients participating in the study that the provision of piped music should be considered as an intervention within the context of ICU services. The rationale for this suggestion is that music therapy has the potential to produce pleasant sensations and experiences (Figure 1)

I wasn't thinking about anything or anyone, I was just focused on me and the song they were singing to me, I was listening to the guitar and I was getting more and more relaxed. (PAT3)

Furthermore, music therapy engenders the perception that the healthcare team is composed of benevolent individuals, which can enhance confidence and reassurance. Similarly, this therapy engages ICU patients who find themselves in unexpected situations. Music therapy generates both a pleasant feeling in them and an improvement in their ability to adapt to coping with their illness.

I think music therapy can have a huge influence on the comfort of being here, it gives you more joy. You think that the people there are working, and you are more alone, so it brings a feeling of warmth and that you are not alone. The music therapist played the song that touches my heart, I felt more accompanied, less sad. (PAT13)

The participant discusses the potential of music therapy as a form of distraction for patients during their hospitalisation and throughout their illness process in the ICU. Furthermore, the patients report that these sessions serve as a way of accompanying them in their lives and bringing them closer to their families and the healthcare staff who attend them. This helps to establish a therapeutic relationship with the healthcare professionals and is identified as a way of bonding with their families.

I was thinking about the sounds of music, what was coming through my ear was music, I was listening to music, thinking about music and I wasn't thinking about anything else, that is, I wasn't thinking about my wife, only about what I was listening to. (PAT4)

This section explores the positive impact of music therapy in the ICU, highlighting its role in humanising care and offering emotional comfort to patients and families. Patients suggested extending the session duration and expanding the hospital‐wide therapy due to its therapeutic value. Music therapy fosters feelings of well‐being, connection with healthcare staff and improved coping during critical illness.

### 6.2 Finding Emotional Balance Through Music Therapy

In this context, patients report that the music therapy session has helped them to manage feelings of discomfort and loneliness in an environment such as the Intensive Care Unit.

There is one thing that is clear, that if I am listening to something and I like it, I fall asleep, I was listening to that and I was extremely happy, it transported me to another place and I fell asleep. (PAT6)

I was thinking about the sounds of the music, what was coming through my ear was music and I wasn't thinking about anything else. (PAT4)

Patients, due to the distress caused by their illness and the environment in which they are treated, may be more or less receptive to the use of music therapy as a complement to other treatments. This can influence them in a positive way, helping them to relax during their illness or the specific ailments they are suffering from. Patients report that music therapy helps them focus their attention on the music in the face of the challenges they face, providing them with motivation to keep fighting. However, they also acknowledge that not everyone benefits equally from this therapy, as individuals' receptiveness varies depending on their physical and mental states. While many feel that music therapy positively affects their mood, some informants shared more mixed experiences. These reflections highlight the subjective nature of music therapy, underscoring that the effectiveness of the therapy may be influenced by the emotional and physical state of the individual at the time.

When you're alone and can only hear the beeps of monitors and machines, music can help you relax and feel calmer. (PAT3)

I like it. I'm more relaxed and I can't hear any noise, just a little music. I think it can help me improve. (PAT5)

The patients discuss the significance of music therapy as a means of alleviating physical discomfort and enhancing their psycho‐emotional well‐being, providing them with tranquillity and calm, without necessarily focusing on the circumstances surrounding them.

It clears your mind from thinking about other things that you think about, it takes your mind off things. (PAT9)

This theme highlights how music therapy helps ICU patients manage discomfort, loneliness and emotional distress, offering moments of calm and distraction. While many reports have positive effects such as relaxation, improved mood and motivation, others note that its impact depends on their physical and emotional state at the time. The findings emphasise the subjective nature of music therapy and its varying reception among individuals.

### 6.3 Coping and Recovering From Illness Through Music Therapy

This theme examines the efficacy of music therapy in alleviating discomfort experienced by patients in the ICU. The patients report that music therapy has served as a coping mechanism for dealing with their illness and their recovery. They have found that music therapy allows them to escape from their current situation and to recall memories of their childhood and youth.

I believe that music therapy influences recovery. For me, music is already important in my life, so, in my life for better or worse, in health and in illness. (PAT7)

It is evident that music therapy has assisted patients in combating the illness they were experiencing at the time of the session.

Patients report that music therapy evokes feelings of well‐being and serves as an incentive for them to persevere during and after their illness. Furthermore, they acknowledge that the music therapy session in the ICU has endowed them with the fortitude to confront their recovery in a more efficacious manner.

Without music I am nobody. The way of playing, the sonority, encouraged me that you have to be positive, raise your head and go up […]. When I'm feeling a bit down, I'll put it on to cheer me up. (PAT3)

This sub‐theme also addresses the distress experienced by patients when admitted to a specialised unit such as the ICU. They describe the experience as challenging and indicate that music therapy can facilitate relaxation and a sense of escape from the situation they are going through. They further highlight the tedious nature of the time spent in the ICU.

I don't know, like a children's game. Or the feeling, when I pick up the tambourine, I said how my grandchildren see me, that's the feeling, of a game, to have fun and often forget your sorrows. (PAT10)

I was amazed by the way he sang. This is the best thing that has happened to me in a long time, apart from that it has given me the courage to go on, it has given me the strength to go on. (PAT12)

This section explores how music therapy helps ICU patients cope with illness and recovery by providing emotional relief, evoking positive memories and offering a sense of escape. Patients describe music therapy as a source of motivation, strength, and well‐being during a challenging period. They also highlight its role in easing the emotional burden of ICU admission and making their hospital stay more bearable.

## 7 Discussion

### 7.1 Humanising Care Through Interdisciplinary Approaches in Critical Care

The results of this study indicate that patients utilise music therapy in a variety of contexts, often describing the music therapy sessions as a pleasant experience that serves to distract them from their circumstances. This distraction allows them to focus on themselves and the music they are listening to. According to the participants' accounts, music therapy can enhance the quality of care and comfort experienced by patients and their families during their stay in an ICU. Furthermore, music therapy can be an effective form of distraction and emotional support in the Intensive Care Unit for hospitalised patients in critical situations. Additionally, Martínez‐Pizarro [24] has demonstrated that music therapy has been employed in patients admitted to the ICU as a distraction strategy in critically ill [24]. The use of music has been exhibited to have a beneficial effect on stress, anxiety, pain and discomfort. This has been evidenced in the context of critically ill patients, where it has been employed as a distraction strategy. Furthermore, the application of music tailored to the individual patient's needs has been demonstrated to enhance the condition and well‐being of patients admitted to the ICU [25]. These experiences are tinged by positive aspects derived from the human quality of care provided by health personnel and family support, factors that strengthen the patient [26].

### 7.2 Finding Emotional Balance Through Music Therapy

Conversely, patients have indicated that music therapy is an essential component in inducing a state of relaxation, particularly during the sessions when they are listening to the music. This study did not investigate the influence of music therapy on sedation and analgesia. However, some patients reported a reduction in pain while undergoing an invasive technique or procedure compared to when they did not have a music therapy session. These findings are in alignment with the conclusions presented by Huang's team [27], which indicated that 90% of the music therapy studies demonstrated statistically and clinically significant improvements in the outcome variables. Among the studies reviewed, 57% supported a reduction in pain and 75% supported a reduction in anxiety [27]. In contrast, Guerra et al. [28] conducted a systematic review of several studies and concluded that there was limited evidence to support or refute the use of music to reduce sedation and analgesia requirements, or to reduce delirium in critically ill adults [28]. Furthermore, Sousa et al. [29] conducted a study on cancer patients in which they observed that music therapy can influence brain waves and frequencies, resulting in the modification of gastric motility. This, in turn, has been shown to be beneficial for the control of nausea and vomiting as side effects of chemotherapy [29]. In conclusion, Erbay Dalli posited that multi‐session music interventions can be employed as a nursing intervention for the management of anxiety levels in ICU patients [30].

### 7.3 Coping and Recovering From Illness Through Music Therapy

The results of this study indicate that the music therapy sessions have served as a source of motivation for the patient participants, facilitating their continued engagement with the illness and recovery process. As Belmonte [31] posits, the use of music exerts a profound influence on motivation, enabling the involvement of patients who, for various reasons, may be less enthusiastic or reluctant towards novel therapeutic activities. These patients may benefit from such activities, which could enhance their health and well‐being [31]. In a further study, Leardi et al. [32] lobbied that patients who were permitted to select their own music to listen to during the surgical procedure exhibited a notable reduction in cortisol levels [32]; this is a potential neuroscientific explanation for the verbalisations observed in this study and in previous studies. Furthermore, Vieyra et al. [33] posit that music can have a calming effect on the nervous system, which helps patients to relax before and during the procedure [33].

## 8 Limitations

It should be noted that this study is subject to a number of limitations.

Communication difficulties during the interviews, particularly with those who were using oxygen masks, presented a challenge. However, this was mitigated by adjusting the audio settings.

The absence of prior qualitative studies on adult ICU music therapy limits the ability to make meaningful comparisons, as the majority of research in this field focuses on paediatric ICUs.

## 9 Implications and Recommendations for Practice

The findings from this study highlight the potential of music therapy as a valuable complementary intervention in the ICU, providing several implications for nursing practice:

1. Music therapy has proven to be an effective tool for humanising the ICU environment and fostering emotional connections between patients, their families and healthcare providers. Nurses can play a pivotal role in facilitating the integration of music therapy into patient care by recognising its potential to alleviate feelings of isolation and enhance emotional well‐being. By supporting the introduction of music therapy sessions, nurses can help create a more comforting and supportive atmosphere that enhances patients' sense of connection and reduces emotional distress.
2. As this study described, music therapy may serve as a powerful relaxation technique, aiding patients in managing the stress and discomfort associated with their illness. Nurses can incorporate music therapy into their care routines by recommending it as a non‐pharmacological strategy for reducing anxiety and promoting relaxation. For patients experiencing physical discomfort or emotional distress, music therapy can offer a mental escape and provide a soothing influence that improves overall psycho‐emotional health. Nurses can further support this practice by ensuring that patients are provided with a personalised music experience tailored to their preferences.
3. Music therapy was also recognised for its role in fostering resilience and improving recovery outcomes. By providing patients with emotional upliftment and a sense of escape from their critical circumstances, music therapy serves as a motivational tool that can inspire patients to face their recovery with renewed strength. Nurses can advocate for the inclusion of music therapy as part of holistic care, promoting its potential to assist patients in their emotional and physical recovery. Encouraging patients to engage in music therapy may enhance their coping mechanisms, enabling them to better manage the challenges of their illness and recovery process.
4. The integration of music therapy into nursing practice offers opportunities for greater interdisciplinary collaboration. Nurses can work closely with music therapists and other healthcare professionals to ensure that music therapy is delivered effectively and consistently. This collaborative approach can help create a more comprehensive care plan that addresses not only the physical needs of patients but also their emotional and psychological well‐being. By incorporating music therapy as part of the overall care strategy, nurses can contribute to a more patient‐centred and holistic approach to ICU care.

## 10 Conclusions

Music therapy emerged as a unique and beneficial intervention, contributing significantly to the humanisation of the ICU environment. Patients perceived the music therapy sessions as memorable events that created a sense of closeness and connection with their families and healthcare staff. The sessions were not only seen as a therapeutic distraction but also as a means of fostering emotional bonds between patients, their loved ones and the healthcare team. Participants expressed that the music made them feel less isolated and more comforted, helping to improve their emotional state and overall sense of well‐being. The experience was described as uplifting, creating a warm atmosphere that alleviated feelings of sadness and loneliness. These findings highlight the need to individualise the moment, ensuring that each session aligns with the patient's emotional and clinical needs, maximising its therapeutic impact.

The findings indicate that music therapy played a crucial role in helping patients relax and manage the distress associated with their illness. Patients reported that the sessions allowed them to disconnect from their environment and focus on the soothing effects of the music, which helped alleviate physical discomfort and reduce emotional distress. Music therapy provided a mental escape, enabling patients to temporarily shift their attention away from their illness, which contributed to an overall improvement in their psycho‐emotional state.

Music therapy was recognised as an effective coping mechanism that supported both emotional and physical recovery. Patients reported that the sessions helped them recall positive memories, which provided a sense of escape from their current critical situation. The emotional upliftment and relaxation induced by music therapy seemed to foster resilience and fortitude, enabling patients to better manage their illness and recovery process. Several patients expressed that music gave them the strength to face their health challenges with renewed optimism, further highlighting its role as a motivational tool in recovery.

## Ethics Statement

The Consolidated Criteria for Reporting Qualitative Research (COREQ) in Appendix 1 has been used [23]. The study was approved by the Ethics Committee of the European University of Madrid (EC 23.238). The study was formally endorsed on 25th April 2023. The authors confirm that they have adhered to the protocols of their institution regarding the publication of participants' data. The right to privacy and informed consent have been respected throughout the course of this study.

## Consent

The authors have obtained informed consent from the patients and/or subjects mentioned in the article, and this document is in the possession of the corresponding author.

## Conflicts of Interest

The authors declare no conflicts of interest.

## Data Availability

The data that support the findings of this study are available on request from the corresponding author. The data are not publicly available due to privacy or ethical restrictions.

## Appendix 1 Consolidated Criteria for Reporting Qualitative Research (COREQ). Modified From Tong et al. [23]

**Table:**

Theme	Guiding question/description	Reported on page
Domain 1. Reflexivity and the research team		
Characteristics of interviewing staff		
1. Interviewer/facilitator	Which author(s) conducted the interviews?	VSO
2. Credentials	What were the Researcher's credentials?	Master in Critical Care
3. Occupation	What was your occupation during the study?	Emergency Nurse
4. Gender	Is the researcher a man or a woman?	Woman
5. Experience and training	What experience does the Researcher have?	14 years as a Nurse in different health care settings
Relationship with participants		
6. Established relationships	Was it an established relationship before the study?	No. Participants who were currently in the Unit were selected.
7. Participant's Knowledge of the Research	What did the participants do that the researcher knows about, for example, personnel, objectives, reasons for doing the research?	They did not know the researcher beforehand
8. Researcher Characteristics	What characteristics were reported about the interviewer? For example, alleged motives and interests in the research topic?	The Nursing Supervisor and the Head of Service informed the participants about the high motivation and interest of the researcher in the subject of a doctoral thesis.
Domain 2. Study design.		
Theoretical framework		
9. Theory and methodological orientation	What methodological orientation underpins the study? For example, grounded theory, discourse analysis, ethnography, phenomenology and content analysis.	Hermeneutic phenomenology
Selection of participants		
10. Sampling	How were participants selected? For example, purpose‐based, convenience, consecutive, snowball.	Purposive and theoretical sampling
11. Selection method	How were participants contacted? For example, face‐to‐face, telephone, email.	Participants were contacted face‐to‐face to explain what the research was about.
12. Sample size	How many participants were in the study?	14
13. Abandonments	How many people dropped out of the study and what reasons did they give?	There were no drop‐outs. 1 participant could not be interviewed due to a worsening health condition.
Adjustments		
14. Adjustments to data collection	Where were the data collected? For example, at home, at the clinic, at your workplace.	In a room set up in the ICU and in the hospitalisation rooms when they were discharged from the ICU, in a quiet, isolated, post‐intervention environment.
15. Presence of non‐participants	Was anyone else present besides the participants and the researcher?	No one else was present. The researcher and the participants at the time of the interviews were alone.
16. Description of the sample	What are the main characteristics of the sample?	PATIENTS: (*n* = 14) 8 women and 7 men. The mean age was 66 years.
Data collection		
17. Interview guide	Were the questions, instructions, guidelines created by the authors? Was it pilot‐tested?	Semi‐structured interviews were conducted following a guide of questions created by the researcher based on the themes of interest identified as emerging in previous similar studies.
18. Repetition of interviews	Were repeated interviews conducted? If so, how many?	No interviews were repeated.
19. Audio/visual recording	Was an audio or visual recorder used to collect the data?	An audio recorder was used for the interviews; videos and photos were used for the sessions.
20. Field notes	Were field notes made during/after the visit?	Field notes were taken during the interview. Similarly, the researcher's notes were subsequently taken on aspects such as method, theoretical content and the course of data collection.
21. Duration	What was the duration of the interviews?	An average of 40 min per interview.
22. Data saturation	Was data saturation discussed?	It was discussed with the directors until data saturation.
23. Return of transcripts	Were the transcripts returned to the participants for comments and/or corrections?	No
Domain 3: Analysis and recommendations		
Data analysis		
24. Number of data encoders	How many data coders coded the data?	6
25. Description of coding	Do the authors provide a description of the coding tree?	Yes, they are provided in the annexes to the thesis.
26. Topic Referral	Are issues decided in advance or derived from the data?	Derived from the data
27. Software	What software, if any, did you use to manage the data?	Atlas‐ti 22
28. Checking of participants	Do participants provide feedback on the findings?	NO
Reports		
29. Submission of quotations	Are participants quoted to illustrate the issues/results?	Participants are cited, with alpha‐numeric codes in each of the citations provided in the report of results.
	Was each quote identified, for example, participant number?	Each appointment was identified with its participant number. Provided in the report of results
30. Consistency between data and results	Is there consistency between the data presented and discussion?	Yes. See results and Discussion
31. Clarity of the main issues	Were the main issues clearly presented in the Outcomes?	Yes, see results
32. Clarity of minor issues	Is there a description and discussion of minor issues?	Yes. Clear description in Results and Discussion of Minor Issues, Sub‐themes
